# Supplementary material for: LORA, Lipid Over-Representation Analysis Based on Structural Information
Source: Anal Chem. 2023 Aug 16;95(34):12600–4. doi: 10.1021/acs.analchem.3c02039 (PMC10469370; doi:10.1021/acs.analchem.3c02039)
Supplement: Supplementary file 1 — ac3c02039_si_001.pdf [file ac3c02039_si_001.pdf]

Supporting Information

LORA, Lipid Over-Representation Analysis Based on Structural Information

Michaela Vondrackova[1], Dominik Kopczynski[2], Nils Hoffmann[3], Ondrej Kuda[4]

[1] Institute of Physiology, Czech Academy of Sciences, Videnska 1083, 14220 Prague, Czechia; orcid.org/0009-0005-6434-0201
[2] Institute of Analytical Chemistry, University of Vienna, 1090 Vienna, Austria; orcid.org/0000-0001-5885-4568
[3] Forschungszentrum Jülich, Institute of Bio- and Geosciences (IBG-5), 52428 Jülich, Germany; orcid.org/0000-0002-6540-6875
[4] Institute of Physiology, Czech Academy of Sciences, Videnska 1083, 14220 Prague, Czechia; orcid.org/0000-0001-7034-4536; corresponding author, Email: ondrej.kuda@fgu.cas.cz

Table of Contents

S1.1 LIPIDOME VISUALIZATION 2

S1.2 COMPARISON OF LORA WITH OTHER SOFTWARE TOOLS 2

S1.3 GOSLIN LIPID STRUCTURAL HIERARCHY 4

S1.4 STATISTICS 5

S1.5 REFERENCES 6

S1.1 Lipidome visualization

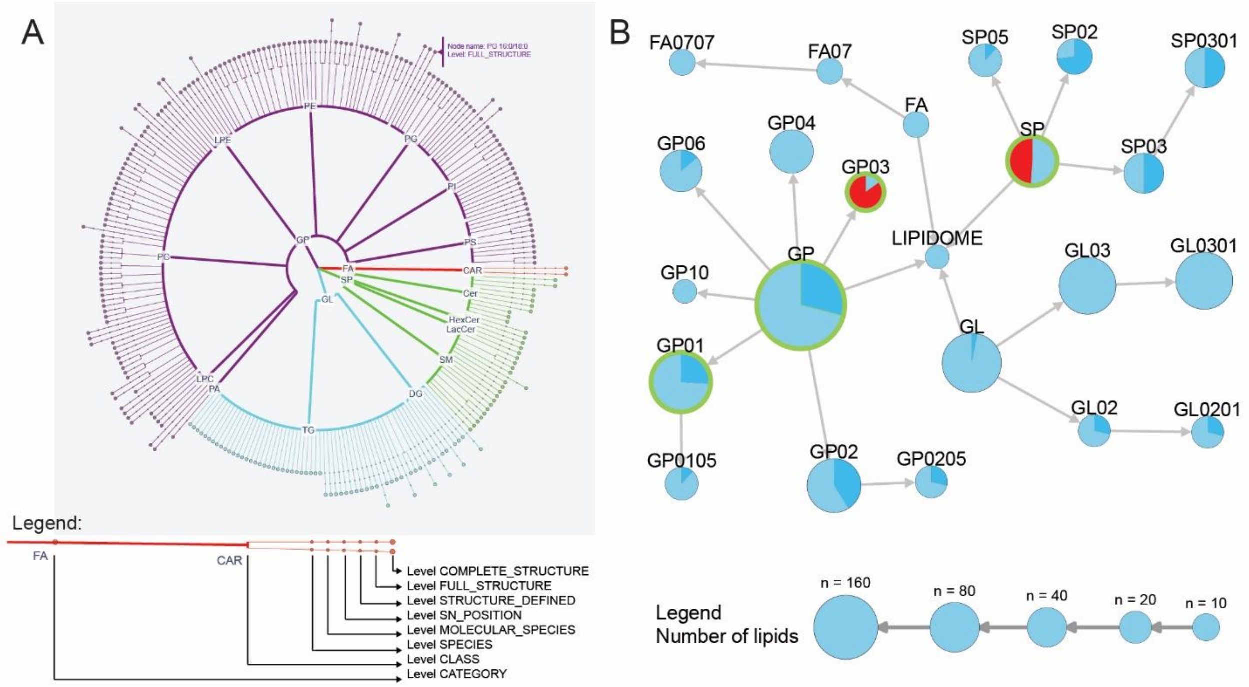

Figure S1: **Lipidome visualization**. A: Hierarchical tree based on Goslin levels for each lipid molecule. B: Lipid network highlighting the number of species and related LORA statistics. Abbreviations of lipid categories, classes and subclasses follow LIPIDMAPS nomenclature and SHORTHAND notation [1].

S1.2 Comparison of LORA with other software tools

Compared to Lipid Mini-On [2], LORA provides more rigorous results by systematically parsing structural information and applying standardized lipid nomenclature rules (see Figure S1A and Table S1). While Lipid Mini-On generates an enrichment network of lipids and terms and stacked bar plots for the classifiers, LORA generates an UpSet plot, a hierarchical lipidome tree, and a structured lipid network with statistically significant values. Therefore, the tools are complementary when provided with a valid list of lipids. In addition, several features improve the performance of LORA over Lipid Mini-On: 1) LORA accepts multiple lipid nomenclature dialects thanks to jGoslin conversion; 2) new LM categories and updates are propagated to LORA via Goslin, and no manual database update is required; and 3) ORA is performed at a specific nomenclature level using all available structural information.

Compared to LION [3], LORA offers the same advantages as Lipid Mini-On, whereas LION contains a hard-coded database of generalized biophysical, chemical, and cell biological properties (MeSH terms) associated with lipids. The main drawback of LION is its outdated database (v. 2020.07.14), which was updated prior to the publication of the Lipid Shorthand Notation.

Enrichment analysis can be performed also using more complex tool for the analysis of lipidomic datasets based on numeric values, group comparison and data processing. *lipidr* generates sets of lipids based on lipid class, total chain length and unsaturation. [4] LipidSig parses the lipid names using an internal algorithm into 'class' and 'subclass' level and general lipid characteristics. Module Differential expression uses two-way ANOVA to

compare ‘characteristics’ (e.g. class, total double bonds, FA length) and ‘Subgroup of characteristics’. Lipid characteristics can be tested also using Fisher's exact test. [5]

Alternatively, bioinformatics tools for ORA of general metabolomics data, such as MetaboAnalyst [6], can be used. However, such tools ignore the lipid structural information and work only with general identifiers.

Epilipidomics datasets containing detailed information about lipid modifications can be pre-processed by LipidLinxX [7] and converted into a nomenclature format compatible with currently supported Goslin grammars.

S1.3 Goslin lipid structural hierarchy

Table S1. Structural hierarchy representation of PE 16:1(6Z)/16:0;5OH[R],8OH;3oxo). LM: LIPID MAPS, HG: Head Group, FA: Fatty Acyl. Adapted from [8]

Level	Name	Description
Category (LM)	Glycerophospholipids (GP)	Lipid category
Class (LM)	Glycerophosphoethanolamine (PE) GP02	Lipid class
Species (LM Subclass)	Phosphatidylethanolamine, PE 32:2;O3	HG, FA summary, two double bond equivalents, three oxidations
Molecular species	PE 16:1_16:1;O3	HG, two FAs, two double bond equivalents, three oxidations
sn-Position	PE 16:1/16:1;O3	HG, SN positions, here: for FA1 at *sn*-1 and FA2 at *sn*-2, two double bond equivalents, three oxidations
Structure defined	PE 16:1(6)/16:1;(OH)2;oxo	HG, SN positions, here: for FA1 at *sn*-1 and FA2 at *sn*-2, three oxidations and unspecified stereo configuration (6) on FA1
Full structure	PE 16:1(6Z)/16:1;5OH,8OH;3oxo	HG, SN positions, here: for FA1 at *sn*-1 and FA2 at *sn*-2, positions for oxidations and stereo configuration (6Z) on FA1
Complete structure	PE 16:1(6Z)/16:0;5OH[R],8OH;3oxo	HG, SN positions, here: for FA1 at *sn*-1 and FA2 at *sn*-2, positions for oxidations and stereo configuration ([R]) and double bond position and stereo configuration (6Z) on FA1

jGoslin version 2.1.0 covers 254 unique lipid head groups across 111 lipid classes, in addition to 74 fatty acyls (FA in LIPID MAPS). Currently, LIPID MAPS contain 47,981 entries, out of which 42,665 entries have a "name" tag. Of these named entries, Goslin can parse 23,764 (55.69%). However, it is important to highlight that not all lipids represented in LIPID MAPS can be expressed using the shorthand nomenclature that Goslin utilizes. For example, certain types such as saccharolipids, ketones, or prenol lipids (accounting for about 14,600 lipids) currently lack a defined and commonly agreed shorthand representation.

S1.4 Statistics

Both Fisher exact and hypergeometric test were performed using functions provided by python package **scipy** (v 1.10.1) suitable for scientific computations.

scipy.stats.fisher_exact(table, alternative='two-sided')

Perform a Fisher exact test on a 2x2 contingency table.
The null hypothesis is that the true odds ratio of the populations underlying the observations is one, and the observations were sampled from these populations under a condition: the marginals of the resulting table must equal those of the observed table. The statistic returned is the unconditional maximum likelihood estimate of the odds ratio, and the p-value is the probability under the null hypothesis of obtaining a table at least as extreme as the one that was actually observed. There are other possible choices of statistic and two-sided p-value definition associated with Fisher's exact test; please see the Notes for more information.

Parameters:

Table: array_like of ints

A 2x2 contingency table. Elements must be non-negative integers.

Alternative: {'two-sided', 'less', 'greater'}, optional

Defines the alternative hypothesis. The following options are available (default is 'two-sided'):
'two-sided': the odds ratio of the underlying population is not one
'less': the odds ratio of the underlying population is less than one
'greater': the odds ratio of the underlying population is greater than one

Returns:

Res: SignificanceResult

An object containing attributes:
Statistic: float
This is the prior odds ratio, not a posterior estimate.
P value: float
The probability under the null hypothesis of obtaining a table at least as extreme as the one that was actually observed.

scipy.stats.hypergeom = <scipy.stats._discrete_distns.hypergeom_gen object>

A hypergeometric discrete random variable.
The hypergeometric distribution models drawing objects from a bin. M is the total number of objects, n is total number of Type I objects. The random variate represents the number of Type I objects in N drawn without replacement from the total population.
As an instance of the *rv_discrete* class, *hypergeom* object inherits from it a collection of generic methods (see below for the full list), and completes them with details specific for this particular distribution.
sf(k, M, n, N, loc=0) -> 1 - cdf

The odds ratio was calculated within the *fisher_exact* function of the scipy library (v 1.10.1). This value was procured as a contingency value alongside the *p*-value. According to scipy's documentation, this is a prior odds ratio, which is usually calculated based on the observed data, not a posterior estimate.

In general, the odds ratio (OR) in Fisher's exact test is calculated as the ratio of the odds of a given event occurring in different groups.

	Event A	Event B
Group 1	a	b
Group 2	c	d

Odds ratio = (a/d) / (c/b)

In the calculations, the contingency table looks like this:

	Query set	Reference lipidome
Enriched	a	b
Not enriched	c	d

S1.5 References

(1) Liebisch, G.; Fahy, E.; Aoki, J.; Dennis, E. A.; Durand, T.; Ejsing, C. S.; Fedorova, M.; Feussner, I.; Griffiths, W. J.; Kofeler, H.; Merrill, A. H., Jr.; Murphy, R. C.; O'Donnell, V. B.; Oskolkova, O.; Subramaniam, S.; Wakelam, M. J. O.; Spener, F. Update on LIPID MAPS classification, nomenclature, and shorthand notation for MS-derived lipid structures. *J. Lipid Res.* **2020**, *61*, 1539-1555.
(2) Clair, G.; Reehl, S.; Stratton, K. G.; Monroe, M. E.; Tfaily, M. M.; Ansong, C.; Kyle, J. E. Lipid Mini-On: mining and ontology tool for enrichment analysis of lipidomic data. *Bioinformatics* **2019**, *35*, 4507-4508.
(3) Molenaar, M. R.; Jeucken, A.; Wassenaar, T. A.; van de Lest, C. H. A.; Brouwers, J. F.; Helms, J. B. LION/web: a web-based ontology enrichment tool for lipidomic data analysis. *Gigascience* **2019**, *8*.
(4) Mohamed, A.; Molendijk, J.; Hill, M. M. lipidr: A Software Tool for Data Mining and Analysis of Lipidomics Datasets. *J. Proteome Res.* **2020**, *19*, 2890-2897.
(5) Lin, W.-J.; Shen, P.-C.; Liu, H.-C.; Cho, Y.-C.; Hsu, M.-K.; Lin, I.-C.; Chen, F.-H.; Yang, J.-C.; Ma, W.-L.; Cheng, W.-C. LipidSig: a web-based tool for lipidomic data analysis. *Nucleic Acids Res.* **2021**, *49*, W336-W345.
(6) Pang, Z.; Chong, J.; Zhou, G.; de Lima Morais, D. A.; Chang, L.; Barrette, M.; Gauthier, C.; Jacques, P. E.; Li, S.; Xia, J. MetaboAnalyst 5.0: narrowing the gap between raw spectra and functional insights. *Nucleic Acids Res.* **2021**, *49*, W388-W396.
(7) Ni, Z.; Fedorova, M. LipidLynxX: a data transfer hub to support integration of large scale lipidomics datasets. *bioRxiv* **2020**, 2020.2004.2009.033894.
(8) Kopczynski, D.; Hoffmann, N.; Peng, B.; Liebisch, G.; Spener, F.; Ahrends, R. Goslin 2.0 Implements the Recent Lipid Shorthand Nomenclature for MS-Derived Lipid Structures. *Anal. Chem.* **2022**, *94*, 6097-6101.
